# Association between Family Support, Stress, and Sleep Quality among College Students during the COVID-19 Online Learning Period

**DOI:** 10.3390/ijerph20010248

**Published:** 2022-12-23

**Authors:** Xiaobing Xian, Yu Zhang, Aiting Bai, Xingpeng Zhai, Hong Hu, Jiao Zhang, Mengliang Ye

**Affiliations:** School of Public Health, Chongqing Medical University, Chongqing 400016, China

**Keywords:** COVID-19, sleep quality, family support, stress, structural equation modeling

## Abstract

(1) Background: During the past 3 years, the COVID-19 pandemic has severely affected the normal school schedule of college students, jeopardizing their mental health, sleep quality, and interpersonal relationships. However, previous studies have focused on the dimension of social support received, and few studies have measured in depth the association of support received from family on adolescents’ physical and mental health. Therefore, this study explored the associations between family support received by Chinese college students during COVID-19 pandemic online classes, stress and sleep quality, and the mediating role of stress. (2) Methods: A cross-sectional study conducted at Chongqing Medical University recruited 712 college students through a university-wide incidental random sample using the Questionnaire Star platform. Statistical description and correlation analysis was conducted using SPSS 25.0, and structural equation modeling was constructed using AMOS 22.0 to test for mediating effects; (3) Results: The family support score of college students during the COVID-19 pandemic online course was 19.41 ± 4.62. Correlation analysis showed that sleep quality was negatively correlated with family support (r = −0.224, *p* < 0.01), positively correlated with stress (r = 0.324, *p* < 0.01), and family support was negatively correlated with stress (r = −0.159, *p* < 0.01). The results of structural equation modeling showed that stress partially mediated the relationship between family support and sleep quality among college students (indirect effect = −0.150, *p* < 0.01, SE = 0.013,95% CI = [−0.208, −0.064]). The model R2 was 36.4%. (4) Conclusions: Schools should consider implementing sleep education, and stress relief curriculum measures to improve the quality of students’ sleep, and should focus on the role that family plays during online classes. This will help students overcome the negative emotional effects of stress in the COVID-19 pandemic and improve their learning efficiency and physical and mental health.

## 1. Introduction

On 30 January 2020, the World Health Organization (WHO) announced that COVID-19 is a “public health emergency of international concern”, which requires international coordination [1]. Under these circumstances, people’s daily lives and normal educational processes are severely affected, especially for students and academics more broadly, creating greater workload and stress [2,3]. In order to reduce the gathering of people and solve the problem of delayed return to school, a new way of learning has emerged—online learning (OL) [4]. Ahrberg K has proposed that academic pressure will affect the sleep quality (SQ) of college students; more than 60% of students reported poor SQ [5,6]. Buysse et al., of the University of Pittsburgh, USA, divided the criteria for monitoring SQ into seven areas: SQ self-assessment, sleep duration, actual sleep duration, sleep efficiency, sleep disorders, daytime dysfunction, and hypnotic drugs [7]. A large number of studies have proved that sleep disorders are closely related to quality of life [8,9,10]; a longitudinal systematic review of nine studies found that insomnia has a bidirectional relationship with anxiety and depression. Sleep duration may affect the development of chronic diseases such as stroke and cancer, and even mortality. Sleep disorder, obesity, hypertension, hyperlipidemia, and other health variables have a U-shaped relationship with sleep duration [11,12,13,14]. Based on the available studies, it is evident that SQ may have an impact on the physical and mental health of college students.

The family is an important bridge to the healthy development and adulthood of adolescents. A review of studies showed that a low quality family environment would increase the pressure faced by teenagers, while emotional and practical support given by the family could reduce the psychological pressure [15,16,17]. In the context of a COVID-19 pandemic, college students are usually unable to return to school normally during the holidays due to an outbreak in the student’s place of residence or school location. Colleges and universities usually choose to direct college students to complete OL tasks at home. Unlike before when they had daily contact with their classmates and teachers at school, during OL, the only people they have daily contact with are their families. The family serves as a protective factor to mitigate the damage caused by stress [18]. Additionally, family identity, environment, behavior, and structure can have an impact on SQ [19,20,21]. Currently, there is no clear definition of family support (FS). Some researchers consider FS as a part of social support [22]. Cross-sectional clinical studies have used structural equation modeling (SEM) to demonstrate that social support can influence SQ [23]. Li et al., showed in a cross-sectional study that adolescents who communicate regularly with their parents are likely to have better sleep quality, and Ye et al., showed in a large sample study in China that improving family cohesion was an effective intervention to alleviate sleep problems among college students during the COVID-19 pandemic. It is clear that the support received from family is important for sleep quality regulation among college students [24,25].

During the COVID-19 pandemic, more and more attention has been paid to the problem of students’ stress [9]. Stress refers to the “wear and tear” of our body when adapting to the changing environment. It has an impact on our body and emotions, and can have a positive or negative impact. On the one hand, stress forces us to take action. On the other hand, stress can lead to feelings of rejection, anger, and depression, leading to health problems such as headaches, rashes with upset stomachs, insomnia, etc. [26]. Studies have shown that there is a significant correlation between high levels of stress and poor SQ. Among students with stress, 86% had poor SQ. The probability of poor SQ of students with high stress level is about 24 times higher than that of students with low stress level [27,28]. Stress can lead to sleep problems such as waking up early and having trouble sleeping [29]. Medical students suffer from poor SQ, such as excessive daytime sleepiness and sleep deprivation, which may be closely related to the heavy academic pressure of medical students [28,30,31]. Undoubtedly, the epidemic of COVID-19 makes college students suffer from this new source of stress during OL [10,32,33]. In summary, stress has a direct impact on SQ. However, in the context of the epidemic, there is a lack of research on the effect of family dimensions on SQ among college students participating in OL, and it is unknown whether stress mediates the relationship between FS and SQ as a mediating effect. Therefore, this study focused on using SEM to understand the relationship between FS, stress, and SQ among college students in the context of OL during the COVID-19 pandemic. We improve the SQ of college students participating in OL at both individual and group levels to provide a scientific basis for relevant policies and theories. Based on the above studies, we propose the following hypotheses.

1. College students who receive higher FS are more likely to have good SQ and lower stress.
2. College students with less stress are prone to have better SQ.
3. College students with higher FS have less stress, which will lead them to have better SQ.

## 2. Materials and Methods

### 2.1. Participants and Procedure

This study recruited 712 current university students from Chongqing Medical University within one week of the end of the home network teaching activities during the COVID-19 epidemic (19 September 2022 to 27 September 2022). Students had been asked to return to school to complete their studies during this period. Questionnaire data containing general demographic characteristics, reviewing psychological stress status, FS received, and SQ during the past month of online classes were collected through a school-wide convenience sampling (episodic sampling). Before participants formally filled out the questionnaire, we had a brief introduction by a trained professional to obtain the knowledge and consent of the participants. The questionnaire filling platform and the data collection and aggregation use the Questionnaire Star software. Inclusion criteria were college students in their sophomore year and above (because freshmen had just passed the college entrance exams and had not started online teaching activities during this period), and exclusion criteria were no history of major illnesses, chronic illnesses, trauma, and incomplete questionnaire responses. In total, 660 samples were included in this study.

### 2.2. Measurement

#### 2.2.1. Basic Demographic Variables

All participants were asked to provide complete questionnaire information, including gender, age, grade, height, weight (height and weight were used to calculate BMI), family economic status (monthly household income), permanent family location, whether they were an only child, and GPA of the previous academic year. The specific questions and option codes are displayed in Appendix A Table A1.

#### 2.2.2. Family Support

FS was assessed using the Perceived Social Support Scale (PSSS) [34], which was developed by Blumenthal et al., in 1987. The scale was translated and modified to form a Chinese version by Qianjin Jiang et al. Participants’ perceptions of support from family members were assessed by using four questions in the FS section of the PSSS, and detailed information on the measurement questions and option assignments is presented in Appendix A Table A1. Existing research indicates that the PSSS has good reliability in Chinese populations [35], with an internal consistency coefficient of 0.83 for the FS component of the scale and a Cronbach’s alpha of 0.886 for the FS component of this study. Higher scores indicate higher levels of received FS.

#### 2.2.3. Stress

The Depression Anxiety Stress Scale 21 (DASS-21) was used to measure participants’ perceived psychological stress over the past month, and the scale is now widely used to measure the current status of depression, anxiety, and stress in children, adolescents, and older adults [36,37]. The stress scale component consists of seven questions, each scored on a four-point scale, and detailed information is presented in Appendix A Table A1, with higher participant scores indicating greater stress. Stress subscale scores greater than or equal to 14 indicate that the current state of stress should be taken seriously. The Cronbach’s alpha for both sides of stress in this study was 0.851.

#### 2.2.4. Sleep Quality

The Pittsburgh Sleep Quality Index (PSQI) scale, developed by Buysse et al. at the Pittsburgh Medical Center in 1989, was translated into Chinese by Xianchen Liu et al., in 1996, and was used to assess the SQ of subjects in the last month [38,39]. There are 18 self-assessment items and 7 components in this scale. Each component has 0–3 points, the cumulative score of each component is the total score of PSQI, the total score range is 0–21 points, and the higher the score means the poorer the SQ, according to the previous experience. In this study, >5 is considered as having sleep disorder [40]. The Cronbach’s alpha for this component in this study was 0.837.

### 2.3. Statistical Analysis

SPSS 25.0 software was used for statistical analysis of the data, and normality tests were performed for the measurement data. Independent samples *t*-test, one-way ANOVA, and C × 2 column χ^2^ test were used for one-way comparisons, and Pearson correlation analysis was used for correlation tests. Mediation effects were modeled by AMOS 22.0 software to construct SEM and validated by Bootstrap method with a set sampling number of 200. The test level was α = 0.05.

## 3. Results

### 3.1. Basic Characteristics Description

A total of 660 college students enrolled in Chongqing Medical University were included in this study, including 473 (71.7%) female students, 287 (43.5%), 273 (41.4%), 56 (8.5%), and 44 (6.7%) sophomores, juniors, seniors, and above, respectively. Furthermore, 444 (67.6%) had a BMI in the normal range. More than half (55.8%) of the students reported a monthly household income of less than 5000 yuan. More than half (57.4%) of the students’ families live in urban areas. The percentage of only children in the family was 30.9%. The detection rate of group stress in the sample was 19.7%, and the percentage of the sample with SQ problems was 47.3%, as shown in Table 1.

### 3.2. Comparison of Differences in Family Support, Stress, and Sleep Quality among College Students in Different Sociodemographic Characteristics Groups

FS scores differed significantly (*p* < 0.05) by family income, home address, GPA in the previous semester, and whether they were only children. SQ was significantly different between genders (*p* < 0.05), and detailed results are presented in Table 1.

### 3.3. Correlation Analysis of Family Support, Stress, and Sleep Quality

The results of Pearson correlation analysis showed that SQ of college students was negatively correlated with FS (r = −0.224, *p* < 0.01), positively correlated with stress (r = 0.324, *p* < 0.01), and FS was negatively correlated with stress (r = −0.159, *p* < 0.01). Other dimensional correlations are shown in Table 2.

### 3.4. Mediating Effect of Stress in Family Support and Sleep Quality

**Figure 1:**
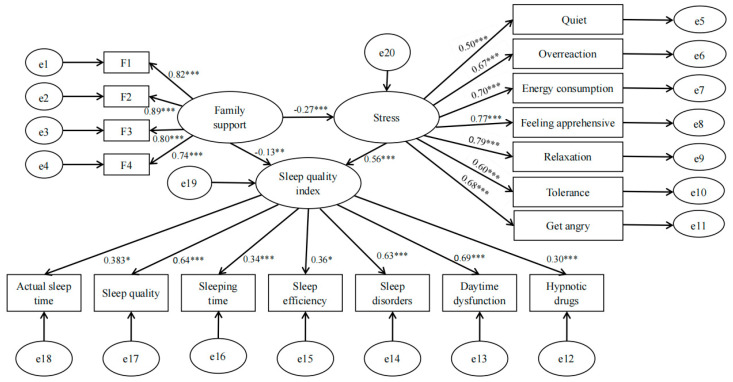
Structural equation model diagram of family support, stress, and sleep quality index. Note: *: *p* < 0.05; **: *p* < 0.01; ***: *p* < 0.001. e1~e20 is residual error.

Based on the correlation between FS, stress, and SQ, we hypothesized that there is a mediating effect of stress in the prediction of SQ by FS. A SEM with FS as the predictor variable (X), stress as the mediating variable (M), and SQ as the outcome variable (Y) was constructed (Figure 1). The model was tested to fit well with χ^2^/df = 3.520 (standardized to less than 5), root mean squared error of approximation (RMSEA) = 0.062 (standardized to less than 0.08), standardized root mean square residual (SRMR) = 0.036 (less than 0.05, which we consider a good model fit), incremental fit index (IFI) = 0.923, adjusted goodness of fit index (AGFI) = 0.906, Tucker–Lewis index (TLI) = 0.911, normative fit index (NFI) = 0.900, comparative fit index (CFI) = 0.923, goodness-of-fit index (GFI) = 0.928 (criterion is greater than 0.90), parsimony fit index (PGFI) = 0.716, adjusted normative fit index (PNFI) = 0.773 (criterion is greater than 0.50). The factor loadings of each significant variable on the corresponding latent variables were significant, indicating that the model was acceptable. The results showed that family support negatively predicted SQ (β = −0.13, P < 0.01) negatively predicted stress (β = −0.27, *p* < 0.001) after controlling for psychological flexibility; stress positively predicted SQ (β = 0.56, *p* < 0.001) after controlling for FS. The mediating effect prediction model was Y= −0.271X + e1; M = −0.27X + e2; and Y= −0.13X + 0.56M + e3 (e1, e2, and e3 represent the regression residuals from the three regressions, respectively).

The bias-corrected Bootstrap method was used to test the mediating effect of stress between FS and SQ, and the 95% CIs for the total, direct, and indirect effect coefficients did not contain 0, indicating that stress partially mediates the relationship between FS and SQ, with a mediating effect of −0.150. The model R2 was 36.4%. The results are displayed in Table 3.

## 4. Discussion

In recent years the prevalence of COVID-19, the SQ, and psychological stress of college students have received much attention from society. Having good SQ and appropriate stress is the “ballast stone” for the healthy growth of adolescents. Based on the research data of college students in Chongqing Medical University, this study confirmed the effect of FS on sleep quality and stress in college students in medical universities, and explored the mediating mechanism of stress between FS and SQ. The results of this study have implications for the promotion of “Healthy China” and the improvement of physical and mental health of medical college students.

The sample of seniors in our study is smaller in the senior year and above, mainly because students above the senior year did not participate in this survey because they were busy preparing for exams or were not in school due to the fact that they were nearing graduation and preparing to participate in further education or internship work. However, this group of students tends to show higher levels of career insecurity and experience greater work intensity later in college [41], so we should pay more attention to their physical and mental health in subsequent research and policy development. The large variation in the composition of the sample, in terms of whether they are only children or not, may be due to the implementation of the one-child policy in China in early 1979, and therefore a large proportion of adolescents in present-day China do not have siblings [42]. During the COVID-19 pandemic OL period, college students received a FS score of 19.41 ± 4.62. Comparison of FS scores based on monthly family income, home address, whether only child or not, and average GPA in the school year showed significant differences between individuals (*p* < 0.05), while there were no significant differences in gender, grade, and BMI. As the initial and most important place for individual growth, the material environment and living atmosphere of the family have an important influence on adolescents’ psychological perceptions. During home-based OL, the study and living room become classrooms, higher income families can provide better material conditions for students, and families living in urban areas have access to relatively rich social resources compared to those living in rural areas, thus showing higher scores for FS [43]. Individuals from one-child families grow up in an environment conducive to self-development and with quality educational resources, and they have more opportunities to receive support from their families than those with siblings. Academic achievement is often used as a measure of whether a person is good or not, and having excellent grades is more likely to result in recognition and good resources that facilitate access to FS [44].

Our study found a negative association between FS and SQ, i.e., higher access to FS by college students was associated with better SQ. It has been argued that FS is part of social support and studies have demonstrated the significant impact of social support on SQ [45,46]. However, for the student population, the social level of awareness and exposure is in its formative stages, so FS still dominates within the support individuals receive. Through correlation analysis we found that the support given by family showed significant negative correlations in all six dimensions of the Pittsburgh Sleep Quality Index evaluation (SQ self-assessment, sleep duration, actual sleep duration, sleep disorders, daytime dysfunction, and hypnotic drugs), which shows that it is quite important to consider FS as a protective factor for college students’ SQ regulation, and the authorities and platforms should strengthen the promotion of positive family concepts so as to improve the SQ problems of students studying online during the COVID-19 pandemic.

We also explored how FS could not only directly affect SQ problems among college students, but also indirectly moderate SQ problems by affecting stress levels. The results showed that FS was negatively related to stress, and stress and SQ were positively related. Stress partially mediated the relationship between FS and SQ. Previous studies have demonstrated the association between having higher levels of stress and poorer SQ among college students [47,48]. FS is demonstrated by the fact that children can communicate with their parents about the difficulties they encounter and receive some emotional support and material resources to solve their problems through their families, thus eliminating the burden of stress and further improving their SQ problems.

Although distance learning and epidemic prevention measures may provide more opportunities to improve SQ, prolonged use of electronics and reduced outdoor activity by college students may affect SQ and biorhythms [33]. During an epidemic, college students face the stress of irregular study, rest and SQ, disrupted rhythm of life, and mental health during a pandemic. In order to maintain internal homeostasis in the presence of stressors, a complex series of responses, including the endocrine, nervous, and immune systems [49,50,51], need to be activated, collectively referred to by scholars as the stress response. The presence of stress increases cortisol, adrenaline levels [52], and the adrenal pathway also has an important role in regulating the 24-h sleep-wake cycle [53]. Prolonged stress levels are associated with hyperactivity of the adrenal pathway, shorter sleep duration, and reduced deep and rapid eye movement sleep, leading to poorer SQ and poorer emotional regulation, which in turn leads to more stress. During the COVID-19 pandemic online study period, college students’ sleep patterns were disrupted, stress levels, SQ and sleep duration were impaired, and our study suggests that FS has a moderating effect on these impairments.

## 5. Conclusions

This study shows that all of our research hypotheses hold true, namely that FS can significantly influence stress levels and SQ among medical college students, and that college students with higher stress levels have worse SQ, and that we find that mediating mechanisms of stress regulation are also significantly present. This study showed that nearly half of college students reported poor SQ and roughly one in five reported stress during COVID-19 pandemic online classes. It suggests that schools, families, and individuals may need to work together to improve the SQ and mental health of college students during pandemic online classes. First, schools should pay attention to the SQ and mental health of students forced to study online at home during the pandemic by adequately scheduling study and rest time, enhancing sleep education, improving psychological support systems, and intervening to treat students with serious psychological problems in a timely manner. In addition, college students should adjust their mindset, fully participate in outdoor sports, develop regular habits of work and rest, and improve their psychological coping skills by establishing and cultivating close relationships with parents and maintaining emotional connections to maintain good mental health.

## 6. Limitations

Although all the hypotheses in this study were tested, this study also contains some limitations. First, this study used a cross-sectional research method, which could not determine the causal relationship between variables. Therefore, longitudinal studies should be conducted in future studies to further discuss the impact mechanisms. Second, the results of this study were based on a medical university, and due to the specificity of the medical student population, generalization of the findings to various college student populations requires further expansion of the representative sample. Third, recall measures of SQ, stress, and FS during online classes were taken within a week after starting regular classes on campus for the previous month, and this inevitably led to some recall bias. In addition, due to the special nature of students in their senior year and above, facing the pressure of further education or employment, the sample content in our study is small, so we should consider focusing on the physical and mental health problems of this group of college students in future studies.

## Figures and Tables

**Table 1:** Comparison of family support, stress, and sleep quality levels among different demographic and sociological characteristics (n = 660).

Variable	n (%)	Family Support Scores ($\bar{x}$ + s)	t/F (*p*)	Stress			Sleep Quality		
				No Stress n (%)	Stress n (%)	χ^2^ (*p*)	Normal n (%)	Abnormal n (%)	χ^2^ (*p*)
Gender			4.719 (0.051)			0.064 (0.80)			5.537 (<0.05)
Male	187 (28.3)	18.85 ± 5.14		149 (79.7)	38 (20.3)		102 (54.5)	85 (45.5)	
Female	473 (71.7)	19.63 ± 4.38		381 (80.5)	92 (19.5)		210 (44.4)	263 (55.6)	
Grade			1.150 (0.328)			1.535 (0.674)			4.613 (0.202)
Sophomore year	287 (43.5)	19.50 ± 4.63		226 (78.7)	61 (21.3)		133 (46.3)	154 (53.7)	
Junior year	273 (41.4)	19.36 ± 4.69		221 (81.0)	52 (19.0)		139 (50.9)	134 (49.1)	
Senior year	56 (8.5)	18.54 ± 4.33		45 (80.4)	11 (19.6)		20 (35.7)	36 (64.3)	
Above	44 (6.7)	20.2 ± 4.48		38 (86.4)	6 (13.6)		20 (45.5)	24 (54.5)	
BMI			1.466 (0.232)			0.365 (0.833)			0.269(0.874)
<18.5	140 (21.2)	18.89 ± 4.64		112 (80.0)	28 (20.0)		64 (45.7)	76 (54.3)	
18.5~23.9	444 (67.6)	19.48 ± 4.63		355 (80.0)	89 (20.0)		213 (48.0)	231 (52.0)	
>23.9	76 (11.2)	19.96 ± 4.50		63 (82.9)	13 (17.1)		35 (46.1)	41 (53.9)	
Monthly family income			−4.679 (<0.001)			0.010 (0.919)			0.357 (0.550)
≤5000	368 (55.8)	18.67 ± 4.65		295 (80.2)	73 (19.8)		188 (51.2)	180 (48.8)	
>5000	292 (44.2)	20.34 ± 4.42		235 (80.5)	57 (19.5)		162 (55.5)	130 (44.5)	
Home addresses			−3.944 (<0.001)			2.116 (0.146)			0.084 (0.772)
Township	281 (42.6)	18.59 ± 4.61		233 (82.9)	48 (17.1)		131 (46.6)	150 (53.3)	
City	379 (57.4)	20.01 ± 4.54		297 (78.4)	82 (21.6)		181 (47.8)	198 (52.2)	
Only child			3.391 (<0.01)			0.654 (0.414)			0.009 (0.924)
Yes	204 (30.9)	20.31 ± 4.58		160 (78.4)	44 (21.6)		97 (47.5)	107 (52.5)	
No	456 (69.1)	19.00 ± 4.59		370 (81.1)	86 (18.9)		215 (47.1)	241 (52.9)	
Performance points			−2.576 (<0.05)			1.294 (0.255)			1.382 (0.240)
<3.0	226 (34.2)	18.77 ± 4.67		187 (82.7)	39 (17.3)		127 (56.2)	99 (43.8)	
≥3.0	434 (65.8)	19.74 ± 4.57		343 (79.0)	91 (21.0)		223 (51.4)	211 (48.6)	
Total	660 (100)	19.41 ± 4.62		530 (80.3)	130 (19.7)		350 (53.0)	310 (47.0)	

**Table 2:** Correlation analysis of family support, stress, and sleep quality.

Variable	Family Support ①	Stress ②	Sleep Quality Index ③	Sleep Quality Self-assessment ④	Sleeping Time ⑤	Actual Sleep Time ⑥	Sleep Efficiency ⑦	Sleep disorders ⑧	Daytime Dysfunction ⑨	Hypnotic Drugs ⑩
① Stress	−0.159 **	1								
② Sleep Quality	−0.224 **	0.324 **	1							
③ Sleep Quality Self-assessment	−0.183 **	0.231 **	0.751 **	1						
④ Sleeping Duration	−0.116 **	0.175 **	0.714 **	0.575 **	1					
⑤ Actual Sleep Duration	−0.099 *	0.093 *	0.528 **	0.207 **	0.160 **	1				
⑥ Sleep Efficiency	−0.002	0.024	0.368 **	0.076	0.155 **	0.384 **	1			
⑦ Sleep Disorders	−0.143 **	0.260 **	0.614 **	0.441 **	0.367 **	0.100 *	−0.005	1		
⑧ Daytime Dysfunction	−0.217 **	0.384 **	0.696 **	0.465 **	0.335 **	0.198 **	0.002	0.468 **	1	
⑨ Hypnotic Drugs	−0.159 **	0.101 **	0.393 **	0.226 **	0.174 **	0.173 **	−0.008	0.227 **	0.175 **	1

Note: *: *p* < 0.05 ; **: *p* < 0.01. ①~⑩ indicate variable numbers

**Table 3:** Mediating effect of stress on the relationship between family support and sleep quality (n = 660).

Family Support	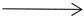	Sleep Quality	S.E.	*p*	β	95%CI
Total effects			0.006	*p* < 0.01	−0.277	(−0.310~−0.190)
Direct effects			0.006	*p* < 0.01	−0.127	(−0.291~−0.015)
Indirect effects			0.013	*p* < 0.01	−0.150	(−0.208~−0.064)

Note: S.E. is the standard error; β is the standardized path coefficient; 95%CI is 95% confidence interval.

## Data Availability

The data sets used and analyzed in this study are available from the corresponding author upon request.

## Appendix A

**Table A1:** Measurements of the variables in this study.

Variables	Definition	Measurements	Options
Basic information survey		Gender	0 female 1 male
		Grade	1 Sophomore 2 Junior 3 Senior 4 Grade Five of University 5 Postgraduates (including doctoral students)
		BMI	1 Less than 18.5 2 From 18.5 to 23.9 3 More than 23.9
		Are you an only child?	1 Yes 2 No
		Your permanent home address	0 Village, town 1 County town, urban district
		Monthly household income	1 Less than 3000 RMB 2 From 3000 to 5000 RMB 3 From 5000 to 10,000 RMB 4 More than 10,000 RMB
		What is your average GPA of all required courses in the last academic year?	1 Less than 2.0 2 From 2.0 to 3.0 3 More than 3.0
Family Support Scale (PSSS)		My family can help me practically and concretely	1 Strongly disagree 2 Disagree 3 Slightly disagree 4 Neutrally 5 Slightly agree 6 Agree 7 Strongly agree
		I can get emotional help and support from my family when I need	1 Strongly disagree 2 Disagree 3 Slightly disagree 4 Neutrally 5 Slightly agree 6 Agree 7 Strongly agree
		I can talk about my problems with my family	1 Strongly disagree 2 Disagree 3 Slightly disagree 4 Neutrally 5 Slightly agree 6 Agree 7 Strongly agree
		My family is willing to assist me in making all kinds of decisions	1 Strongly disagree 2 Disagree 3 Slightly disagree 4 Neutrally 5 Slightly agree 6 Agree 7 Strongly agree
Stress Scale (DASS-21)		I find it hard to keep quiet.	0 Never 1 Sometimes 2 Often 3 Always
		I often have overreaction to things. (Overreacting, overly sensitive).	0 Never 1 Sometimes 2 Often 3 Always
		I feel like I used up a lot of energy.	0 Never 1 Sometimes 2 Often 3 Always
		I feel uneasy.	0 Never 1 Sometimes 2 Often 3 Always
		I find it hard to relax myself.	0 Never 1 Sometimes 2 Often 3 Always
		I can’t tolerate anything that prevents me from continuing my work.	0 Never 1 Sometimes 2 Often 3 Always
		I found myself easily get angry.	0 Never 1 Sometimes 2 Often 3 Always
The Pittsburgh Sleep Quality Index		sleep quality self-assessment	0 Very good 1 good 2 bad 3 Very bad
		Sleep duration	0 0 1 From 1 to 2 2 From 3 to 4 3 From 5 to 6
		Actual sleep duration	0 More than 7 h 1 From 6 to 7 h 2 From 5 to 6 h 3 Less than 5 h
		Sleep efficiency	0 More than 85 % 1 From 75 to 84 % 2 From 65 to 74 % 3 Less than 65 %
		Sleep disorders	0 0 1 From 1 to 9 2 From 10 to 18 3 From 19 to 27
		Hypnotic drugs	0 None 1 Less than once a week 2 From once to twice a week 3 More than three times a week
		Daytime Dysfunction	0 0 1 From 1 to 2 2 From 3 to 4 3 From 5 to 6
